# List prices and clinical value of anticancer drugs in China, Japan, and South Korea: a retrospective comparative study

**DOI:** 10.1016/j.lanwpc.2024.101088

**Published:** 2024-05-16

**Authors:** Jay Pan, Xiaolin Wei, Hao Lu, Xueer Wu, Chunyuan Li, Xuelian Hai, Tianjiao Lan, Quanfang Dong, Yili Yang, Mihajlo Jakovljevic, Jing Zhou

**Affiliations:** a HEOA Group, West China School of Public Health and West China Fourth Hospital, Sichuan University, Chengdu, China; b Institute for Healthy Cities and West China Research Center for Rural Health Development, Sichuan University, Chengdu, China; c Dalla Lana School of Public Health, University of Toronto, Toronto, Canada; d School of Public Health, Imperial College London, London, UK; e Nanjing Municipal Center for Disease Control and Prevention, Nanjing, China; f Department of Hematology, Peking University Third Hospital, Beijing, China; g UNESCO - The World Academy of Sciences (TWAS), Trieste, Italy; h Shaanxi University of Technology, Hanzhong, China; i Department of Global Health Economics and Policy, University of Kragujevac, Kragujevac, Serbia

**Keywords:** Anticancer drugs, Prices, Clinical value, China, Japan, South Korea

## Abstract

**Background:**

High prices of anticancer drugs have raised concerns due to their financial impact on patients and healthcare systems. This study aimed to assess the initial and latest list prices and clinical value of reimbursed anticancer drugs in China, Japan, and South Korea.

**Methods:**

We identified anticancer drugs newly approved by the National Medical Products Administration of China from January 2012 to June 2022, and by the Pharmaceuticals and Medical Devices Agency of Japan and the Ministry of Food and Drug Safety of South Korea up until June 2022. We compared initial and latest treatment prices between countries and assessed clinical value using patients’ survival, quality of life (QoL), and European Society for Medical Oncology Magnitude of Clinical Benefit Scale (ESMO-MCBS). We calculated Spearman rank correlation coefficients of treatment prices with clinical value for individual countries and employed regression analyses to investigate whether the relationship between prices and clinical value was modified by the country setting.

**Findings:**

Our cohort included 91 anticancer drug indications, with 60 listed for reimbursement in China, 91 in Japan, and 87 in South Korea. Median treatment prices were highest in Japan, followed by South Korea, and lowest in China, both for initial prices (US$64082 vs. US$45529 vs. US$19144, p < 0.0001) and latest prices (US$50859 vs. US$31611 vs. US$18666, p < 0.0001). Over time, China (*β* = −0.047, p < 0.0001) and South Korea (*β* = −0.049, p < 0.0001) witnessed more significant price reductions compared to Japan (*β* = −0.013, p = 0.011). The correlations between both initial and latest treatment prices and clinical value (QoL and ESMO-MCBS) were more significant and stronger in China and South Korea than in Japan, although Japan exhibited slightly stronger correlations in terms of survival compared to China and South Korea. The relationship between clinical value and treatment prices may not be modified by the country setting.

**Interpretation:**

In comparison, South Korea’s list prices and their correlations with clinical value appear reasonable. Policymakers in Japan could enhance efficiency by controlling prices and aligning them with clinical value, while China would need to take substantial steps to expand anticancer drug coverage.

**Funding:**

10.13039/501100001809National Natural Science Foundation of China (72374149 and 72074163), and China Center for South Asian Studies, 10.13039/501100004912Sichuan University.

**Research in context**

**Evidence before this study**

High prices and the mismatch between prices and the clinical value of anticancer drugs are critical global issues. Previous studies primarily focused on investigating prices and their correlation with clinical value of anticancer drugs within the US and European countries, including the UK, France, Germany, and Italy. Although attention has been directed towards China and Japan, evidence from East Asia regarding this issue remains insufficient. Moreover, there is a lack of international comparative research on this subject involving countries outside the US and Europe. For this study, we identified anticancer drugs newly approved by the NMPA in China from January 2012 to June 2022, and by the PMDA in Japan and the MFDS in South Korea up until June 2022. We compared initial and latest list prices among the three countries and calculated Spearman rank correlation coefficients to assess the correlations between treatment prices and clinical value (patients’ survival, QoL, and ESMO-MCBS) for each individual country. We then employed regression analyses to investigate whether the relationship between prices and clinical value was modified by the country setting.

**Added value of this study**

This is the first study to compare treatment prices based on both initial list prices and latest list prices, as well as their relationships with the clinical value of reimbursed anticancer drugs in East Asia. Japan had the highest initial and latest treatment prices, followed by South Korea, and China had the lowest. Furthermore, China and South Korea have experienced more substantial price reductions over time than Japan. However, the correlations between both initial and latest treatment prices and clinical value (QoL and ESMO-MCBS) were more significant and stronger in China and South Korea than in Japan, although Japan exhibited slightly stronger correlations in terms of survival compared to China and South Korea.

**Implications of all the available evidence**

While South Korea’s list prices and their correlation with clinical value appear reasonable, policymakers in Japan could reexamine their pricing strategies to control prices and enhance their alignment with the clinical value, and China would need to take substantial steps to expand coverage of anticancer drugs.

## Introduction

The escalating and considerable worldwide cancer burden, accompanied by unmet clinical needs, has propelled the booming R&D of anticancer treatments over the past decades.^1^^,^^2^ In 2022, the global expenditure on anticancer drugs has reached $196 billion, with projections indicating a further ascent to $375 billion by the end of 2027.^3^ Given the noteworthy financial impact for both patients and healthcare systems, which subsequently manifests as reduced patient access, diminished adherence, and ultimately compromised health outcomes, high prices of anticancer drugs are extensively criticized.^4^

Countries have been struggling to provide coverage for more innovative drugs within constrained budgets. The largest economies and major oncology drug markets in East Asia, namely China, Japan, and South Korea, have implemented comprehensive strategies within their social health insurance systems to mitigate drug prices while striving to maximize value for money.^5^ In South Korea, the positive list system and price negotiations based on Health technology assessment (HTA) have been implemented for new drugs since 2007.^6^ Similarly, China formally adopted reimbursement-linked price negotiations informed by HTA for new or exclusive drugs since 2017.^7^^,^^8^ In contrast, Japan’s approach to pricing new drugs involves pricing rules rather than negotiations. Specifically, the cost calculation system is employed for new drugs without existing comparable drugs, while benchmark pricing is used for new drugs with existing comparators.^9^ Premiums may be applied, predominantly to account for innovation, alongside factors such as usefulness and marketability. If manufacturers have no complaints about the pricing plan, the drug will be listed for reimbursement. After the initial list prices were formed in the three countries, price cuts may apply under certain rules.^6^ Additional details on the pricing and reimbursement of new drugs in the three countries can be found in the Appendix.

There are major concerns about the clinical value of anticancer drugs other than prices. As indicated by previous studies, the magnitude of the clinical value of anticancer drugs varies widely, and an appreciable proportion of drugs offer no to little added benefits over existing drugs.^10^ Rationalizing the relationship between resource inputs and outcomes can improve the efficiency of countries’ health systems. Under such context, the possibility of the misalignment between prices and clinical value of anticancer drugs has been highlighted as an underlying issue leading to resource misallocation. Consequently, health authorities’ efforts in optimizing value for money are under scrutiny.

Previous studies have addressed this issue particularly within the context of the US and European countries.^11^^,^^12^ Although attention has been paid to China and Japan in East Asia, these studies were grounded in either list prices or latest prices.^13^, ^14^, ^15^ Price changes of reimbursed anticancer drugs after being listed can be substantial, potentially altering the relationship between prices and clinical value. Furthermore, measures of prices and clinical value vary across existing studies, limiting the ability to compare across countries. International comparative studies among countries with similar or different pricing mechanisms, which enable stakeholders to examine the effectiveness and rationality of their pricing strategies, have not yet been conducted in East Asia. In this study, we aimed to analyze the initial list prices, latest list prices, and clinical value of reimbursed anticancer drugs in China, Japan, and South Korea.

## Methods

### Data sources and extraction

A list of anticancer drugs newly approved in China between January 2012 and June 2022 was identified through the National Medical Products Administration (NMPA) and with reference to the work by Yichen et al.^15^ In this list, we further identified drugs that had also been approved by the Ministry of Food and Drug Safety (MFDS) in South Korea and the Pharmaceuticals and Medical Devices Agency (PMDA) in Japan up until June 2022.^16^, ^17^, ^18^ We finally restricted our study cohort to the same drug–indication pairs that have been approved by all three of the aforementioned agencies. In this study cohort, we identified the initial listed indications for reimbursement and those listed as of June 2023 (including both initial and supplementary listed indications) in China (National Healthcare Security Administration, NHSA), Japan (Ministry of Health, Labour, and Welfare, MHLW), and South Korea (Health Insurance Review & Assessment Service, HIRA). The specific indication definitions may differ in the three countries, in which case the defined commonalities would be extracted with reference to the work by Trotta et al., to inform later analysis and to ensure comparability among China, Japan, and South Korea.^19^

To assess the clinical value of included indications, we extracted overall survival (OS), progression-free survival (PFS), and quality of life (QoL) from pivotal randomized controlled phase III or phase II clinical trials at initial listing and the latest time. To identify the same pivotal clinical trial in the three countries for each indication, we consulted and compared the review reports and drug labels issued by the NMPA in China, the PMDA in Japan, and the MFDS in South Korea. In cases where multiple trials were relevant, we sequentially chose the trial that best matched the commonalities of the indications in the three countries, targeted Asian population, or had the best clinical outcome.^11^^,^^12^ We also used the validated European Society for Medical Oncology Magnitude of Clinical Benefit Scale (ESMO-MCBS) to assess their clinical value at initial listing and the latest time. The latest ESMO-MCBS scores for solid tumors were obtained from the ESMO website and scores for hematological malignancies were obtained from the published work of ESMO Working Group.^20^^,^^21^ The ESMO website updates ESMO-MCBS Scores regularly. Where the latest ESMO-MCBS scores were not available for indications, we assessed the scores based on latest evidence from clinical trials using ESMO-MCBS evaluation forms after studying the online tutorials. We also applied this approach to assess initial ESMO-MCBS scores, considering clinical evidence available at the time of the initial listing. ESMO-MCBS scores A or B in the curative setting and 5 or 4 in the non-curative setting were considered clinically meaningful benefits.^20^

We extracted the initial and latest (as of June 2023) list prices for initial listed indications, as well as the latest list prices for those listed indications as of June 2023 in our sample for Japan (MHLW) and South Korea (HIRA) in July 2023.^22^^,^^23^ In China, the prices were retrieved from relevant documents issued by the NHSA and were complemented by searching a Chinese pharmaceutical database during the corresponding period.^24^ The initial and latest treatment prices for corresponding indications over expected treatment durations were estimated based on respective list prices and dosing information from drug labels. The expected treatment durations for these indications were the median treatment durations reported in pivotal clinical trial publications. In cases where these publications did not provide median treatment durations, we referred to drug labels as a supplementary source. For drugs that required dosing based on body weight or body surface area (BSA), we assumed commonly used standard patient values of 70 kg and 1.7 m^2^, respectively, in line with previous studies.^4^^,^^11^^,^^12^ When multiple treatment prices could be calculated (i.e., multiple drug strengths), we prioritized the strength common to all three countries. In cases where the strengths differed across countries, we selected the strength that resulted in the lowest treatment prices.^12^ We converted initial and latest treatment prices from national currencies to US dollars using the 2022 exchange rates, both with and without adjustment for purchasing power parity (PPP). The exchange rates and PPP were obtained from OECD.Stat, the Organisation for Economic Co-operation and Development’s statistical database. To adjust initial treatment prices for inflation, we extracted monthly inflation data from the OECD.Stat.

Two researchers with backgrounds in pharmacy independently extracted these data, and consensus was reached through discussion in cases of disagreement. This study did not require institutional review board approval because it was based on publicly available information.

### Statistical analysis

We used Kruskal–Wallis tests to compare the initial and latest treatment prices for initial listed indications, as well as the latest treatment prices for indications listed as of June 2023, in China, Japan, and South Korea. For initial listed indications within each country, we employed simple linear regression analysis to analyze the percentage changes in prices from the date of listing to June 30, 2023, considering the associated durations.

We computed survival benefits as the absolute differences in median OS between the experimental and control groups. In cases where OS data was unavailable, we evaluated survival benefits based on the absolute differences in median PFS between the experimental and control groups.^8^^,^^25^ The QoL was categorized into three categories: improvement, no difference, and reduction or unavailable, after reviewing the relevant contents in the publications for the pivotal trials.^8^^,^^26^^,^^27^ As for the clinical value measured by the ESMO-MCBS, high benefit was defined as a score of A–B in the curative setting or 4–5 in the non-curative setting, and low benefit was defined as any other score.^11^

We calculated Spearman rank correlation coefficients between both initial and latest treatment prices and clinical value (survival in either OS or PFS, QoL, and ESMO-MCBS) for corresponding indications within each country. Additionally, we employed multiple linear regression analyses to investigate the association between each measure of clinical value and the percentage changes in treatment prices across all three countries. Given the relationship between clinical value and treatment prices may be modified by the country setting, we considered interaction effects (model specification can be found in the Appendix). We applied linear regressions with log-transformed outcome data since our dependent variables all had skewed distributions.

We conducted three sensitivity analyses to assess the robustness of our results. First, we used OS as the measure of survival instead of the aggregated survival in either OS or PFS. Second, we repeated the analyses of correlations between treatment prices and clinical value after restricting the sample to indications listed in all three countries. Third, we employed generalized estimating equations (GEE) to examine the relationships between treatment prices and clinical value within each country, as well as across countries. The GEE was used because our analyses were conducted at the indication level, and it accommodates the presence of multiple indications for anticancer drugs.

All data were collected using a pre-designed Excel file and were imported into R (version 4.1.0) for statistical analyses. The ggplot2 (version 3.3.5) was used for visualization. Spearman’s correlation coefficients and regression coefficients were reported with 95% confidence intervals (95% CI).^28^^,^^29^ p values of less than 0.05 were considered statistically significant.

### Role of the funding source

The funders of the study had no role in study design, data collection, data analysis, data interpretation, or writing of the report.

## Results

**Fig. 1:**
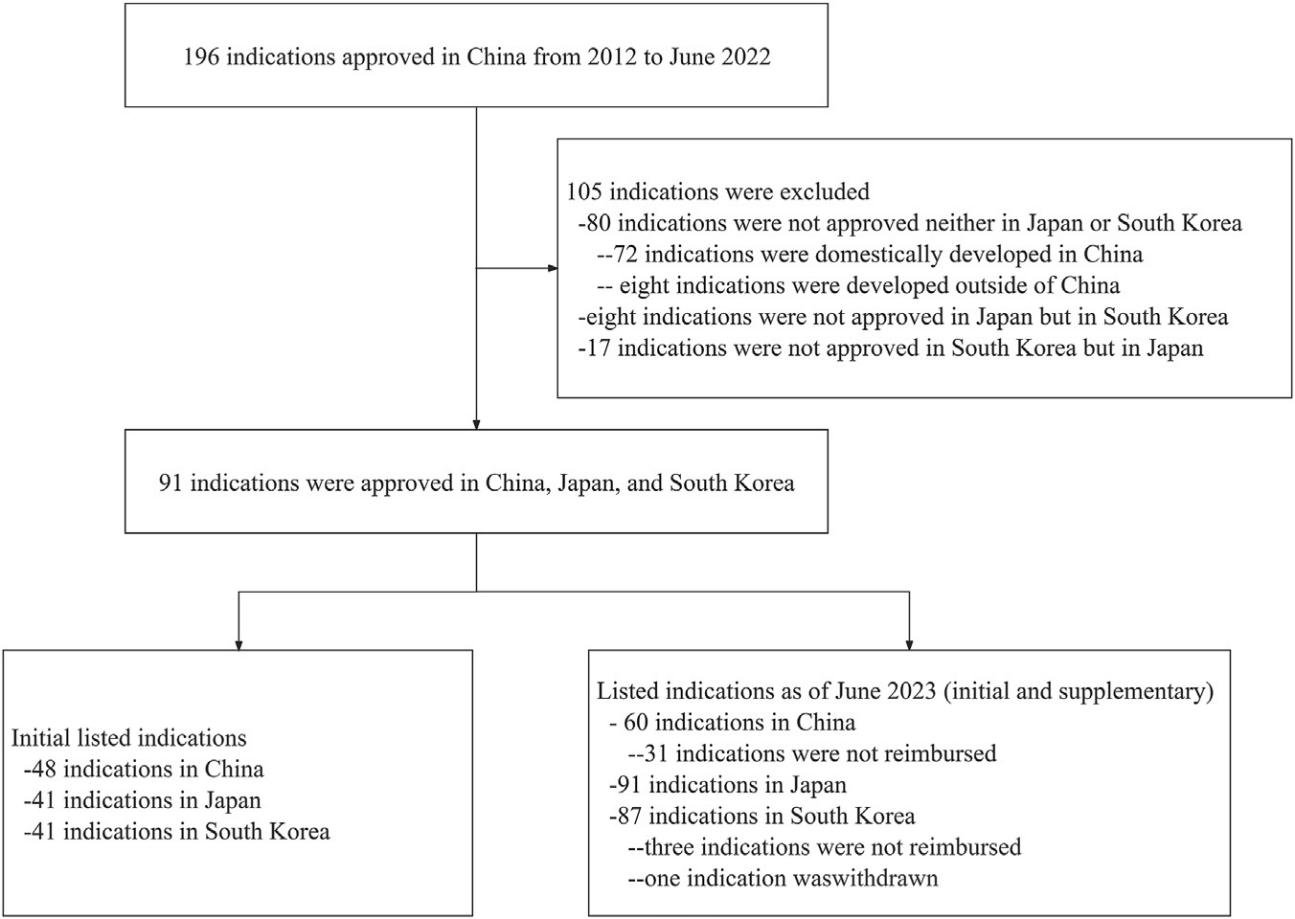
**Flowchart of sample selection**.

Our study cohort included a total of 91 indications that were approved in China, Japan, and South Korea (Fig. 1 and Table 1). Of these, 22 (24%) were approved for hematological cancers, while the remaining 69 (76%) were approved for the treatment of solid tumors. The most common solid tumors were lung cancer (16 [18%]), breast cancer (11 [12%]), and prostate cancer (nine [10%]). Indications from targeted small molecule accounted for the most (43 [47%]), followed by immunotherapy (22 [24%]) and targeted monoclonal antibody (14 [15%]). Seven (8%) indications were for curative intent, while 84 (92%) were for palliative intent. Pivotal studies for these indications were mostly randomized controlled trials (81 [89%]), with ten being single-arm clinical trials. Within the cohort of 91 indications, there were 48 (53%) initial listed indications for reimbursement in China, 41 (45%) in Japan, and 41 (45%) in South Korea. By June 2023, a total of 60 (66%), 91 (100%), and 87 (96%) indications (including both initial and supplementary listed indications) in China, Japan, and South Korea, respectively, were listed for reimbursement.

**Table 1:** Characteristics of indications in the study sample.

Indications approved in China, Japan and South Korea (N = 91)	Number	Percent
Cancer site		
Hematological	22	24
Lung	16	18
Breast	11	12
Prostate	9	10
Other	33	36
Drug type		
Targeted small molecule	43	47
Immunotherapy	22	24
Targeted monoclonal antibody	14	15
Hormonal agent	8	9
Chemotherapy	4	4
Treatment setting		
Curative	7	8
Palliative	84	92
Trial type		
Randomized controlled trial	81	89
Single-arm clinical trial	10	11

Reimbursement status in the three countries (N = 91)	Number	Percent
Initial listed indications		
China	48	53
Japan	41	45
South Korea	41	45
Listed indications as of June 2023		
China	60	66
Japan	91	100
South Korea	87	96

### Initial and latest treatment prices in China, Japan, and South Korea

**Fig. 2:**
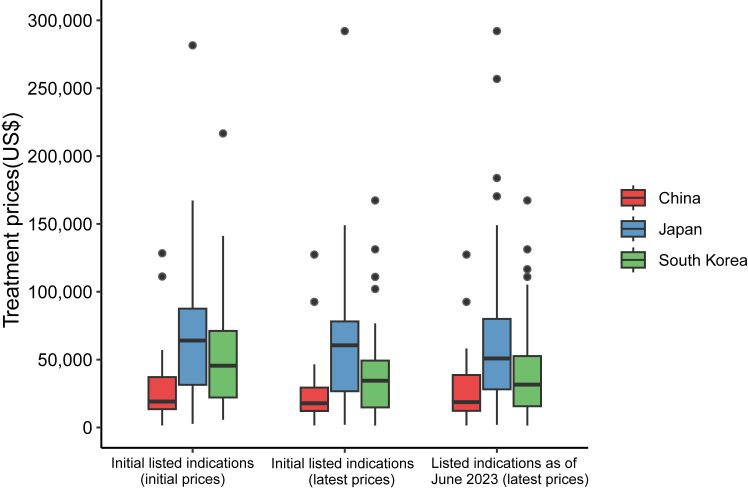
**The initial and latest treatment prices of corresponding indications in China, Japan, and South Korea.** Notes: Price information was available for all included listed indications in individual countries (initial listed indications: N = 48 in China, N = 41 in Japan, and N = 41 in South Korea; listed indications as of June 2023: N = 60 in China, N = 91 in Japan, and N = 87 in South Korea). Of note, because one indication was withdrawn from the market in South Korea, the analysis for the latest prices in South Korea excluded this indication. The box displays the median and interquartile range (IQR). The band near the middle of the box is the median, and the bottom and top of the box are the 1st and 3rd quartiles (the 25th and 75th percentiles, respectively). The solid lines below and above the box describe the bottom and top whiskers. The small dots indicate extreme outliers, which are located outside 1.5 times the interquartile range above the upper quartile.

**Fig. 3:**
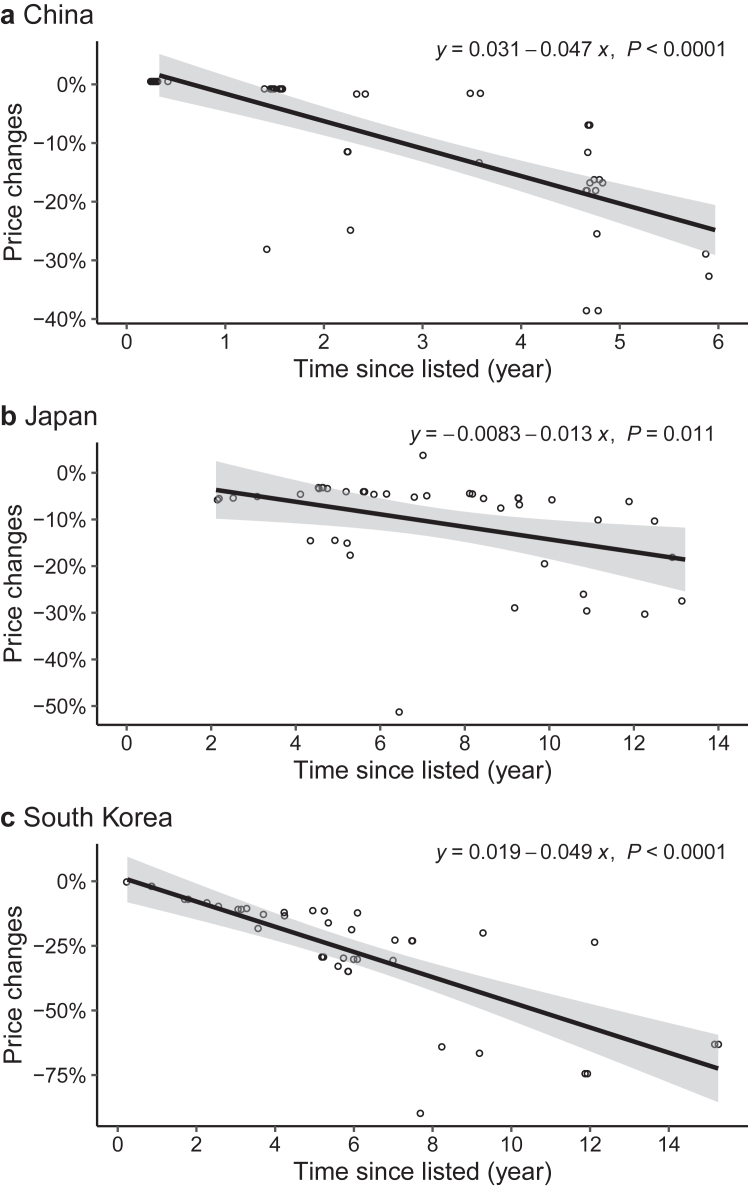
**Treatment price changes over time for initial listed indications in China, Japan, and South Korea.** Notes: All initial listed indications in respective countries were included in this analysis, except one indication that was withdrawn in South Korea (N = 48 in China, N = 41 in Japan, N = 41 in South Korea). Each dot represents an initial listed indication in its respective country. The dots are accompanied by a fitted curve (line) and 95% CIs (shaded area).

The median initial treatment prices for initial listed indications differed significantly among the three countries: US$19144 (IQR: 13,490–37,691) in China, US$64082 (IQR: 31,420–87,584) in Japan, and US$45529 (IQR: 22,117–71,188) in South Korea (p < 0.0001) (Fig. 2, left side). The latest treatment prices of these initial listed indications have been decreased to US$17865 (IQR: 12,075–29,470) in China, US$60553 (IQR: 26,720–78,140) in Japan, and US$34484 (IQR: 14,428–52,656) in South Korea as of June 2023 (p < 0.0001) (Fig. 2, middle). Price reductions were more substantial with longer durations from the listing date to June 2023 in the three countries (Fig. 3). Specifically, China (*β* = −0.047, p < 0.0001) and South Korea (*β* = −0.049, p < 0.0001) experienced greater price reductions over time compared to Japan (*β* = −0.013, p = 0.011). When including supplementary listed indications (those listed as of June 2023), the median latest treatment prices were US$18666 (IQR: 12,231–38,916), US$50859 (IQR: 28,125–80,002), and US$31611 (IQR: 15,630–52,602) in China, Japan, and South Korea (p < 0.0001), respectively (Fig. 2, right side).

Similar results were observed when restricting the sample to listed indications that were common across all three countries and using PPP-adjusted prices for comparison. The initial treatment prices for initial indications in Japan (PPP$ 80,148 [IQR: 43,268–114,113]) were 1.24 times (IQR: 1.03–1.47) those of South Korea (PPP$71768 [IQR: 22,230–98,506]) and 2.20 times (IQR: 1.80–3.40) those of China (PPP$33797 [IQR: 22,573–59,967]) (p = 0.0011). For indications listed as of June 2023, the latest treatment prices were PPP$87578 (IQR: 40,205–115,613) in Japan, PPP$52507 (IQR: 21,318–94,117) in South Korea, and PPP$32223 (IQR: 20,623–65,881) in China (p < 0.0001), making the latest treatment prices in Japan 1.48 times (1.07–1.97) and 2.40 times (1.70–2.90) those of South Korea and China, respectively.

### Correlations of treatment prices with clinical value in China, Japan, and South Korea

**Fig. 4:**
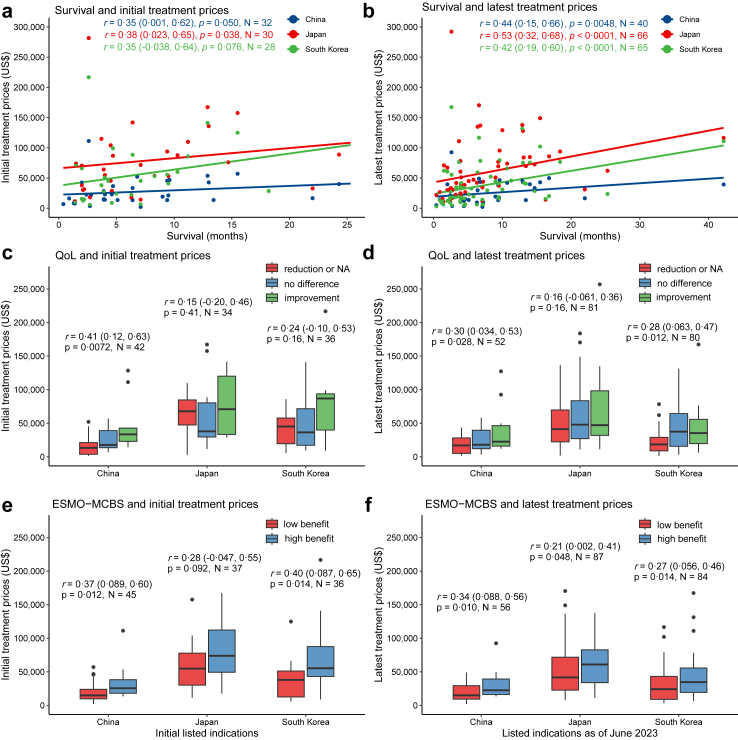
**Correlations of treatment prices with clinical value for listed indications in China, Japan, and South Korea.** Notes: For each analysis, we excluded indications with missing required data on clinical value. The left side of the figure shows the correlations between initial treatment prices and initial clinical value (survival, QoL, ESMO-MCBS) for initial listed indications with Spearman’s correlation coefficients *r* (95% confidence interval) and asymptotic p values and sample sizes N, while the right side shows the correlations between latest treatment prices and latest clinical value for listed indications as of June 2023 in China, Japan, and South Korea. For (a) and (b), dots with different colors represent indications in different countries. Survival: the absolute differences in median overall survival or progression-free survival between the experimental and control groups. For (c), (d), (e) and (f), the box displays the median and interquartile range (IQR). The band near the middle of the box is the median, and the bottom and top of the box are the 1st and 3rd quartiles (the 25th and 75th percentiles, respectively). The solid lines below and above the box describe the bottom and top whiskers. The small dots indicate extreme outliers, which are located outside 1.5 times the interquartile range above the upper quartile.

Indications with any clinical value data available were included for prices and value analyses (Data availability for clinical value and for each analysis [Supplementary Tables S2–S4] can be found in the Appendix). Initial treatment prices for initial listed indications were moderately correlated with initial clinical value in China (survival: *r* = 0.35, 95% CI [0.001, 0.62], p = 0.050; QoL: *r* = 0.41, 95% CI [0.12, 0.63], p = 0.0072; ESMO-MCBS: *r* = 0.37, 95% CI [0.089, 0.60], p = 0.012) (Fig. 4, left side). While Japan exhibited one moderate and significant correlation for survival (*r* = 0.38, 95% CI [0.023, 0.65], p = 0.038), correlations for QoL (*r* = 0.15, 95% CI [−0.20, 0.46], p = 0.41) and ESMO-MCBS (*r* = 0.28, 95% CI [−0.047, 0.55], p = 0.092) were weak and insignificant. In South Korea, a moderate and significant correlation was observed for ESMO-MCBS (*r* = 0.40, 95% CI [0.087, 0.65], p = 0.014), but not for survival (*r* = 0.35, 95% CI [−0.038, 0.64], p = 0.076) and QoL (*r* = 0.24, 95% CI [−0.10, 0.53], p = 0.16). These correlations became more significant in the three countries based on latest treatment prices and latest clinical value, after accounting for changes in prices and clinical value (Supplementary Table S5).

When including supplementary listed indications (those listed as of June 2023), the correlations of latest treatment prices with latest clinical value were all moderate and significant in China (survival: *r* = 0.44, 95% CI [0.15, 0.66], p = 0.0048; QoL: *r* = 0.30, 95% CI [0.034, 0.53], p = 0.028; ESMO-MCBS: *r* = 0.34, 95% CI [0.088, 0.56], p = 0.010) (Fig. 4, right side). These correlations in South Korea were moderate to weak, but all were statistically significant (survival: *r* = 0.42, 95% CI [0.19, 0.60], p < 0.0001; QoL: *r* = 0.28, 95% CI [0.063, 0.47], p = 0.012; ESMO-MCBS: *r* = 0.27, 95% CI [0.056, 0.46], p = 0.014). In Japan, while statistically significant correlations for survival (*r* = 0.53, 95% CI [0.32, 0.68], p < 0.0001) and ESMO-MCBS (r = 0.21, 95% CI [0.002, 0.41], p = 0.048) were observed, the correlations for ESMO-MCBS and QoL (*r* = 0.16, 95% CI [−0.061, 0.36], p = 0.16) were weak.

### Relationship between treatment prices and clinical value among the three countries

In regression analyses with data pooled across all three countries, higher clinical value (survival, QoL, ESMO) generally exhibited a positive association with higher treatment prices for both initial listed indications (Table 2) and those listed as of June 2023 (Table 3). Compared to China, interactions between country and different measures of clinical value were all found to be insignificant in Japan and South Korea, suggesting that the relationship between clinical value and percentage changes in the initial and latest treatment prices may not be modified by the country setting (Table 2, Table 3).

**Table 2:** Regression analyses of initial treatment prices and initial clinical value for initial listed indications in China, Japan, and South Korea.

Variables	log (initial treatment prices)					
	Model (1)		Model (2)		Model (3)	
	Coefficient (95% CI)	p value	Coefficient (95% CI)	p value	Coefficient (95% CI)	p value
Survival	0.018 (−0.004, 0.040)	0.11				
QoL_no difference			0.277 (−0.003, 0.550)	0.047		
QoL_improvement			0.515 (0.182, 0.848)	0.0027		
ESMO_high benefit					0.262 (0.070, 0.456)	0.0080
Japan	0.480 (0.187, 0.774)	0.0016	0.630 (0.334, 0.926)	<0.0001	0.514 (0.319. 0.710)	<0.0001
South Korea	0.170 (−0.145, 0.485)	0.29	0.471 (0.180, 0.726)	0.0018	0.264 (0.057, 0.471)	0.013
Survival # Japan	−0.002 (−0.034, 0.029)	0.88				
Survival # South Korea	0.015 (−0.024, 0.054)	0.45				
QoL_no difference # Japan			−0.272 (−0.684, 0.141)	0.19		
QoL_improvement # Japan			−0.297 (−0.763, 0.171)	0.21		
QoL_no difference # South Korea			−0.219 (−0.614, 0.176)	0.27		
QoL_improvement # South Korea			−0.262 (−0.746, 0.222)	0.29		
ESMO_high benefit # Japan					−0.069 (−0.358, 0.220)	0.64
ESMO_high benefit # South Korea					0.045 (−0.244, 0.335)	0.76

Notes: For each analysis, we excluded indications with missing required data on clinical value. Treatment prices were calculated based on initial prices. Survival: the absolute differences in median overall survival or progression-free survival between the experimental and control groups.

QoL = quality of life, NA = not available. Reference categories: QoL: reduction or NA; ESMO: low benefit; country: China.

**Table 3:** Regression analyses of latest treatment prices and clinical value for listed indications as of June 2023 in China, Japan, and South Korea.

Variables	log (latest treatment prices)					
	Model (1)		Model (2)		Model (3)	
	Coefficient (95% CI)	p value	Coefficient (95% CI)	p value	Coefficient (95% CI)	p value
Survival	0.018 (0.004, 0.032)	0.015				
QoL_no difference			0.214 (−0.038, 0.466)	0.095		
QoL_improvement			0.414 (0.108, 0.721)	0.0083		
ESMO_high benefit					0.249 (0.068, 0.429)	0.0071
Japan	0.371 (0.172, 0.570)	0.00032	0.488 (0.230, 0.746)	0.00025	0.549 (0.192, 0.905)	0.0027
South Korea	0.111 (−0.089, 0.311)	0.27	0.151 (−0.107, 0.409)	0.25	0.182 (−0.179, 0.542)	0.32
Survival # Japan	0.002 (−0.017, 0.020)	0.87				
Survival # South Korea	0.005 (−0.014, 0.025)	0.57				
QoL_no difference # Japan			−0.100 (−0.423, 0.223)	0.54		
QoL_improvement # Japan			−0.204 (−0.589, 0.181)	0.30		
QoL_no difference # South Korea			0.054 (−0.269, 0.377)	0.74		
QoL_improvement # South Korea			−0.104 (−0.491, 0.282)	0.60		
ESMO_high benefit # Japan					−0.109 (−0.339, 0.121)	0.35
ESMO_high benefit # South Korea					−0.026 (−0.258, 0.205)	0.82

Notes: For each analysis, we excluded indications with missing required data on clinical value. Treatment prices were calculated based on latest prices. Survival: the absolute differences in median overall survival or progression-free survival between the experimental and control groups.

QoL = quality of life, NA = not available. Reference categories: QoL: reduction or NA; ESMO: low benefit; country: China.

### Sensitivity analyses

In sensitivity analyses, the results of the relationship between treatment prices and clinical value remained largely consistent when employing OS as the measure of survival (Supplementary Tables S6 and S7), restricting the sample to indications listed in all three countries (Supplementary Tables S8 and S9), and using generalized estimating equations (Supplementary Tables S10–S13).

## Discussion

This is the first study to compare treatment prices based on both initial list prices and latest list prices, as well as their relationships with the clinical value of reimbursed anticancer drugs in East Asia. We found that among the three countries, Japan had the highest initial and latest treatment prices, followed by South Korea, and China had the lowest. Furthermore, China and South Korea have experienced more substantial price reductions over time than Japan. However, the correlations between both initial and latest treatment prices and clinical value (QoL, and ESMO-MCBS) were more significant and stronger in China and South Korea than in Japan, although Japan exhibited slightly stronger correlations in terms of survival compared to China and South Korea.

Ensuring timely and affordable access, safeguarding the sustainability of budgets, and stimulating innovation for therapeutic needs are triple aims that healthcare systems generally pursue.^30^ Our results revealed that China, Japan, and South Korea may have different focal points in terms of pricing and reimbursement when it comes to achieving the triple aims. China, which maintains significantly lower anticancer drug prices than Japan and South Korea, has apparently prioritized affordable patient access and the sustainability of medical insurance funds in alignment with its economic development level. The fact that only 60 out of the 91 included drug indications were listed for reimbursement in China, while all indications were listed in Japan and 87 indications in South Korea, underscores the crucial role of price negotiation informed by HTA in China in shaping pricing and reimbursement decisions based on patient needs and limited resources. From another perspective, China’s practice might negatively impact drug innovation and compromise the coverage of anticancer drugs. Therefore, China could consider further expanding the coverage of anticancer drugs within its limited resources.^31^

We found that both the initial and latest treatment prices of reimbursed anticancer drugs in Japan were the highest compared to those in China and South Korea. Pricing rules in Japan differ significantly from the price negotiation based on HTA implemented in China and South Korea. The bargaining power of health authorities in these two countries has a remarkable impact on drug price reduction with different magnitudes. Moreover, we also found that price reductions over time were the slowest in Japan. After initial listing, drug prices in Japan were adjusted biennially until 2020 and have been adjusted annually since 2021, primarily based on discrepancies between list prices and actual prevailing market prices.^9^ However, evidence shows that most on-patent drugs are not significantly affected by price revisions based on price divergence, and competition between generic drugs or biosimilars and off-patent drugs is limited.^32^^,^^33^ This could partially explain the limited price reduction observed in Japan compared to China and South Korea, where the influence of price regulation and market competition is more prominent in driving price reductions.^34^^,^^35^ While the rationale behind Japan’s highest drug prices may be attributed to incentives for innovation and the economic development level, its financial sustainability is threatened by demographic, economic, and political factors.^36^ Our findings highlight the importance for policymakers in Japan to further address the high prices of anticancer drugs by considering both initial list prices and subsequent price changes.

The alignment of drug prices and clinical value has great potential to benefit patients and healthcare systems from two interrelated dimensions: accounting for the value of drugs and optimizing medical resource allocations.^26^^,^^37^ The economic value of anticancer drugs should therefore reflect the magnitude of health gain to justify price increases, and funds for low-value anticancer drugs should be redirected to more valuable anticancer drugs to maximize health outcomes. For example, drugs whose prices do not match their value are supposed to be subject to lower prices, which enables finite resources to be allocated toward treatments that offer patients greater clinical benefits. Our study employed three different measures (survival, QoL, and ESMO-MCBS) to assess the clinical value of anticancer drugs. The differences in correlations between treatment prices and different measures of clinical value observed among the three countries may be partially attributable to differences in value measurements embedded in the pricing systems. While all three countries emphasize the added value of anticancer drugs compared to existing treatments, their specific implementation approaches vary. In China, the pricing system may have effectively influenced the moderate and statistically significant correlations between anticancer drug prices and all measures of clinical value, as well as the lowest price levels and substantial price reductions observed.^8^ In contrast, Japan exhibited weaker and less significant correlations between both initial and latest treatment prices and clinical value (QoL and ESMO-MCBS), albeit slightly stronger correlations for survival compared to China and South Korea. This means that Japanese policymakers may need to align prices more closely with clinical value at respective stages, in addition to controlling prices. In comparison, the list prices in South Korea and their correlations with clinical value appear reasonable.

Previous studies on the correlations between prices and clinical value of anticancer drugs have primarily focused on initial indications.^11^^,^^12^^,^^26^^,^^38^ To account for price changes and supplementary indications over time, we examined both the initial and latest prices, along with their correlations with clinical value for initial listed indications, as well as the latest prices and their correlations with clinical value for indications listed as of June 2023, in China, Japan, and South Korea. We found that the magnitude and significance of correlations were likely to change following price changes and the listing of supplementary indications. This highlights the necessity of continually monitoring the correlations between prices and clinical value, taking into account market dynamics to some extent.

Making resource allocation decisions according to the respective value of health interventions to maximize health benefits for patients and societies has become a prevalent strategy in many countries.^39^ We found that greater clinical value was generally positively associated with higher treatment prices in the three countries, and we did not find evidence supporting significant differences in this relationship among China, Japan, and South Korea. This means the magnitude of increased clinical value was consistently and similarly reflected in the magnitude of increased prices in China, Japan, and South Korea.

Findings from the US and European countries have demonstrated that treatment prices were either not correlated or weakly correlated with clinical value.^11^^,^^12^^,^^26^ However, sample and methodological differences across studies have limited the ability to make direct comparisons. In particular, the primary differences related to the calculation of treatment costs may partly explain different conclusions observed among studies and countries.^40^ In this study, we considered treatment durations when calculating treatment prices, considering that treatment duration varies across anticancer drugs and indications, making daily prices and monthly prices imprecise measures of the total financial impact from treatment and biasing price and value comparisons.^8^^,^^26^^,^^40^ While international comparative studies on this topic in Asia are limited, future studies are encouraged to build on this analysis by extending it to other Asian countries.

This study has several limitations. First, the survival in either OS or PFS as a value measurement in our study was aggregated.^8^^,^^25^ In cases where OS data in median times was not available, we used added survival in PFS as a surrogate. In addition, one of the categories of QoL was ‘reduction or not available’ due to the low rate of QoL reporting. Evidence showed that the reporting of QoL in clinical trials was associated with positive trial outcomes, while harm was under-reported in clinical trials.^41^ Second, all measures of clinical value originated from pivotal clinical trials, which cannot fully represent the effectiveness of drugs in real-world settings, though they are of great importance to inform pricing and reimbursement decisions.^42^ Future studies could build on this study to incorporate real-world evidence. Third, list prices in this study were at the national level and might not reflect the actual prices paid by individual health insurers or by patients. Fourth, our study did not account for confounding variables that could potentially influence the relationship between prices and value. Last but not least, our findings cannot be extrapolated to individual indications as well as indications that were not supported by the pivotal clinical trials included in our study. Moreover, since our study only included indications that were approved in all three countries, the findings in individual countries may differ when considering all approved anticancer drugs within each country.

### Conclusions

Japan had the highest initial and latest treatment prices, followed by South Korea, and China had the lowest. In contrast, the correlations between both initial and latest treatment prices and clinical value (QoL, and ESMO-MCBS) were more significant and stronger in China and South Korea than in Japan, although Japan exhibited slightly stronger correlations in terms of survival compared to China and South Korea. While South Korea’s list prices and their correlation with value appear reasonable, policymakers in Japan could reexamine their pricing policies to control prices and enhance their alignment with the clinical value, and China would need to take substantial steps to expand and accelerate coverage of anticancer drugs.

## Contributors

JZ and JP conceived the study, interpreted the results, and drafted the manuscript. XLW provided valuable insights and critically revised the manuscript. HL, XEW, CYL, and XLH collected the data. JZ and HL accessed, verified, and analyzed the data. TJL provided constructive input on data analysis. All authors were involved in manuscript preparation and approved the final version for publication.

## Data sharing statement

All the data used in this study are from publicly accessible databases.

## Declaration of interests

We declare no competing interests.
