# Supplementary material for: List prices and clinical value of anticancer drugs in China, Japan, and South Korea: a retrospective comparative study
Source: Lancet Reg Health West Pac. 2024 May 16;47:101088. doi: 10.1016/j.lanwpc.2024.101088 (PMC11107456; doi:10.1016/j.lanwpc.2024.101088)
Supplement: Appendix [file mmc1.docx]

## Appendix

| *Contents* | *Page* |
| --- | --- |
| Pricing and reimbursement for new drugs in China, Japan, and South Korea | 2 |
| Model specification | 5 |
| Table S1. Characteristics of 91 anticancer drug indications approved in China, Japan, and South Korea. | 6 |
| Data availability of clinical value for 91 included indications | 12 |
| Data availability for each analysis | 13 |
| Correlations of initial and latest treatment prices with clinical value for initial listed indications | 16 |
| Robust analysis using OS as the measure of survival instead of using aggregated survival in either OS or PFS | 17 |
| Robust analysis after restricting the sample to indications that were listed in all three countries | 18 |
| Robust analyses using generalized estimating equations (GEE) | 19 |

### **Pricing and reimbursement for new drugs in China, Japan, and South Korea**

**Pricing and reimbursement for new drugs in China**

To address the issue of high drug prices, promote patient access, and ensure the sustainability of the medical insurance fund, China formally launched national reimbursement-linked price negotiation in 2017. Since then, health authorities have been negotiating prices for innovative drugs directly with pharmaceutical companies annually, informed by health technology assessment (HTA), trying to realize value-based strategic purchase of medical insurance (Tang et al., 2020; Liu et al., 2021; Wen et al., 2023). If an agreement on the reimbursement price is reached between the two sides during the negotiation stage, the candidate drug becomes eligible for inclusion in the National Reimbursement Drug List (NRDL). As of January 2023, six rounds of direct price negotiations with pharmaceutical companies have been conducted, resulting in the inclusion of numerous newly negotiated drugs in the NRDL.

The price negotiation in China is still in its early stages and constantly evolving. From 2018 onward, the newly established National Healthcare Security Administration (NHSA) took over the responsibility of the price negotiation from the Ministry of Human Resources and Social Security (MOHRSS). For candidate drugs qualified for negotiation, the NHSA’s target prices are determined by two parallel groups consisting of public medical insurance executives and pharmacoeconomics experts, respectively (Liu et al., 2021). The former estimates the prices based on the pre-negotiation prices and the sustainability of the insurance fund. Meanwhile, the latter focuses on the comparative effectiveness and safety of the candidates compared to existing treatments, and assesses the pharmacoeconomic reports and budget impact analysis submitted by pharmaceutical companies using domestic and international prices as references, in an attempt to align prices more closely with clinical benefits (Liu et al., 2021). After completing expert reviews and assessments, the NHSA then conducted price negotiations with pharmaceutical companies, and the agreed-upon price was established and applied nationwide.

Price cuts for drugs may apply under certain rules after initial listing for reimbursement through price negotiation (NHSA, 2023). The financial impact on medical insurance fund and major changes in market conditions are two important triggers for price adjustment after listing (NHSA, 2023).

References

Tang M, Song P, He J. Progress on drug pricing negotiations in China. *Biosci Trends*. 2020;**13**(6):464-8.

Liu GG, Wu J, He X, Jiang Y. Policy Updates on Access to and Affordability of Innovative Medicines in China. *Value Health Reg Issues* 2022;**30**:59-66

Wen J, Li M, Jiang Y. Cost effectiveness of innovative anti-cancer drugs and reimbursement decisions in China. *Health Policy and Technology* 2023;**12**(2):100742

National Healthcare Security Administration (NHSA). Announcement of the National Medical Security Administration on the "Rules for Negotiating Drug Contract Renewals" and "Rules for Non-Exclusive Drug Bidding". 2023. <http://www.nhsa.gov.cn/art/2023/7/21/art_109_11063.html> (accessed Dec 11 2023).

**Pricing and reimbursement for new drugs in Japan**

In Japan, the pricing of new drugs is regulated by the Ministry of Health, Labour, and Welfare (MHLW) and determined based on pre-established pricing rules rather than through negotiation (Shiroiwa et al., 2017). Drugs must be covered by the NHI drug price standard to be used in insurance-covered healthcare. The NHI Drug Price Standard is an item list that specifies drugs eligible for insurance-covered healthcare and includes a price table indicating reimbursable prices. In Japan, new drugs are listed four times a year (February, May, August, November) within 60 days after receiving marketing approval in principle, and no later than 90 days (Makoto et al., 2018).

The pricing methods for new drugs encompass the Cost Accounting System and the Similar Efficacy Comparison Method (Takayama and Narukawa, 2016; Makoto, 2018). The Cost Accounting System is applicable to new drugs without similar drugs on the market, considering manufacturing costs, selling expenses, research expenses, operating income, marketing costs, and consumption tax to establish a base price (Makoto, 2018). Similar Efficacy Comparison Method is applied to new drugs with similar drugs on the market, setting the base price by referencing the daily treatment cost of similar drugs. Beyond the base prices determined by the Cost Accounting System and the Similar Efficacy Comparison Method, the MHLW establishes premiums based on the drug's innovation (such as mechanism of action, significant efficacy and safety improvements), usefulness (clinical benefits), marketability (such as orphan drugs), pediatric use, and first-time market entry in Japan (Makoto, 2018). These prices may be adjusted after comparison with average prices in the United Kingdom, Germany, France, and the United States. The final price, or the NHI drug price standard, is determined by the MHLW and applied nationwide upon approval by the Central Social Insurance Medical Council. In case of disagreement with the drug pricing, pharmaceutical companies can appeal, and the Drug Pricing Organization under the Central Social Insurance Medical Council will reevaluate the drug's pricing after considering the company's arguments (Takayama and Narukawa, 2016).

To address rising costs of healthcare expenditure in Japan, the MHLW implemented a 3-year pilot HTA program in 2016 (Kamae et al., 2016). In April 2019, pharmacoeconomic requirements for list-price adjustment were institutionalized following provisional implementation of the HTA program 2016–2019. HTA in Japan is designed to aid in determining or adjusting price (Kamae et al., 2016).

After listing, drug prices undergo regular revisions based on an official survey of the actual sales prices (market prices) to medical institutions and pharmacies (Makoto et al., 2018; Maeda et al., 2021). The timing for regular revisions was every two years until 2020 and is now conducted annually from 2021. There are other special rules for price revision, including market expansion-related repricing, repricing for indication change, premium rewards for innovative development, price reduction for long-listed drugs, dosage/regimen change-related repricing, and other calculations at the time of repricing for orphan drugs, pediatric indications, and genuine clinical usefulness (Maeda et al., 2021).

References

Shiroiwa T, Fukuda T, Ikeda S, Takura TJHP. New decision-making processes for the pricing of health technologies in Japan: the FY 2016/2017 pilot phase for the introduction of economic evaluations. *Health Policy* 2017; **121**(8): 836-41.

Makoto. Introduction of Japanese Pricing System. ISPOR Asia Pacific 2018; 2018.

Takayama A, Narukawa M. Pharmaceutical Pricing and Reimbursement in Japan: For Faster, More Complete Access to New Drugs. *Ther Innov Regul Sci* 2016; **50**(3): 361-7.

Kamae I, Thwaites R, Hamada A, Fernandez JL. Health technology assessment in Japan: a work in progress. *J Med Econ* 2020; **23**(4): 317-22.

Maeda H, Okabe A, Sakakura K, Ng DB, Akazawa M. Relationships between developmental strategies for additional indications and price revisions for anticancer drugs in Japan. *BMC Health Serv Res* 2021; **21**(1): 1329.

**Pricing and reimbursement for new drugs in South Korea**

In South Korea, the Drug Expenditure Rationalization Plan (DERP) came into effect in 2007 as a strategic response to mitigate the rising pharmaceutical expenditure in the country (Kwon and Godman, 2017). The DERP comprised two pivotal components: the establishment of the positive list system (PLS), formulated through rigorous cost-effectiveness appraisals, and the implementation of a price negotiation process involving the National Health Insurance Service (NHIS) as the payer and pharmaceutical companies (Kwon and Godman, 2017).

Pricing and reimbursement decisions for new drugs involve a two-step process with the Health Insurance Review and Assessment (HIRA) and the NHIS playing crucial roles (Kim et al., 2021). In accordance with the DERP, these procedures encompass HIRA's decision-making on reimbursements and subsequent pricing negotiations conducted by the NHIS. Initially, HIRA evaluates the potential reimbursement for new drugs, taking into account factors such as clinical usefulness, cost-effectiveness, and budgetary impact (Bae and Lee, 2009; Park et al., 2012; Bae et al., 2016). Following HIRA's determinations on reimbursement, drugs identified as potentially reimbursable undergo face-to-face price negotiations between the NHIS and pharmaceutical companies within 60 days to establish their prices and anticipate the budget impact based on projected utilization (Kwon and Godman, 2017). Throughout the negotiation process, NHIS considers prices in OECD countries, Taiwan, China, Singapore, as well as the prices of substitute drugs within the same or related therapeutic classes (Kwon and Godman, 2017; Kim et al., 2021).

After listing, price cuts are applied under certain rules, including price-volume agreements (PVA), actual transaction pricing (ATP), price cutting with expanded indication, and price cutting of patent-expired originals and generics (Kwon and Godman, 2017; Kim and Lee, 2020).

References

Kwon HY, Godman B. Drug Pricing in South Korea. *Applied health economics and health policy* 2017; **15**(4): 447-53.

Kim S, Cho H, Kim J, Lee K, Lee JH. The current state of patient access to new drugs in South Korea under the positive list system: evaluation of the changes since the new review pathways. *Expert Rev Pharmacoecon Outcomes Res* 2021; **21**(1): 119-26.

Bae EY, Lee EK. Pharmacoeconomic guidelines and their implementation in the positive list system in South Korea. *Value Health* 2009; **12 Suppl 3**: S36-41.

Park SE, Lim SH, Choi HW, et al. Evaluation on the first 2 years of the positive list system in South Korea. *Health Policy* 2012; **104**(1): 32-9.

Bae EY, Hong JM, Kwon HY, et al. Eight-year experience of using HTA in drug reimbursement: South Korea. *Health Policy* 2016; **120**(6): 612-20.

Kim S, Lee JH. Price-Cutting Trends in New Drugs after Listing in South Korea: The Effect of the Reimbursement Review Pathway on Price Reduction. *Healthcare (Basel)* 2020; **8**(3).

### **Model specification**

$${Prices}_{ic}=\beta_{0}+\beta_{1}*{value}_{i}+{\beta_{2}*country}_{c}+\beta_{3}*({value}_{i}*{country}_{c})+\varepsilon_{i}$$

Notes: ${Prices}_{ic}$ represents the treatment prices over the expected treatment duration of the indication *i* in country *c*. In different models, ${value}_{i}$ represents one of the three different measures of clinical value, including survival benefits, quality of life, and ESMO-MCBS. Survival was coded as continuous variable, while quality of life was coded as reduction or NA, no difference, or improvement, and ESMO-MCBS was coded as high benefit or low benefit. Ternary variable $country$ stood for China, Japan, or South Korea. To test the hypothesis that the relationship between changes in clinical value and changes in percentage treatment prices may vary by the country setting, interaction term of country and clinical value is added and is represented by ${value}_{i}*{country}_{c}$. $\beta_{3}$ is the parameter of interest, with a significant value representing the relationship between treatment prices and clinical value is modified by the country setting.

### **Table S1. Characteristics of 91 anticancer drug indications approved in China, Japan, and South Korea.**

| **Generic name** | **Indication** | **Initial listed indication** | **List time, CHN** | **List time, JPN** | **List time, KR** | **Trial number** | **Trial name** | **Trial type** | **Initial prices (US$), CHN** | **Latest prices (US$), CHN** | **Initial prices (US$), JPN** | **Latest prices (US$), JPN** | **Initial prices (US$), KR** | **Latest prices (US$), KR** | **Survival (month)** | **QoL** | **ESMO-MCBS** |
| --- | --- | --- | --- | --- | --- | --- | --- | --- | --- | --- | --- | --- | --- | --- | --- | --- | --- |
| Lorlatinib | NSCLC (first line) | CHN, KR | 2023/3/1 | 2021/11/25 | 2022/9/1 | NCT03052608 | CROWN | randomized | 28403 | 28541 | NA | 73395 | 45685 | 44784 | NA | no difference | high benefit |
| Lorlatinib | NSCLC | CHN, JPN, KR | 2023/3/1 | 2018/11/20 | 2022/9/1 | NCT01970865 | B7461001 | single-arm | 29181 | 29323 | 77884 | 75406 | 46936 | 46011 | NA | NA | high benefit |
| Brigatinib | NSCLC | CHN, JPN, KR | 2023/3/1 | 2021/4/21 | 2019/5/1 | NCT02737501 | ALTA-1L | randomized | 42740 | 42948 | 135854 | 127977 | 86850 | 75223 | 13 | improvement | high benefit |
| Trastuzumab Emtansine | breast cancer | CHN | 2023/3/1 | 2020/8/21 | 2019/8/8 | NCT01772472 | KATHERINE | randomized | 18013 | 18101 | NA | 65039 | NA | 52980 | NA | NA | high benefit |
| Trastuzumab Emtansine | breast cancer (advanced) | CHN, JPN, KR | 2023/3/1 | 2014/4/17 | 2017/8/1 | NCT00829166 | EMILIA | randomized | 14153 | 14222 | 54859 | 51102 | 51223 | 41628 | 4.0 | improvement | high benefit |
| Brentuximab | Hodgkin's lymphoma | CHN, JPN, KR | 2023/3/1 | 2014/4/17 | 2016/2/1 | NCT00848926 | SG035-0003 | single-arm | 19149 | 19242 | 68641 | 64928 | 52831 | 40643 | NA | NA | low benefit |
| Brentuximab | anaplastic large-cell lymphoma | CHN, JPN, KR | 2023/3/1 | 2014/4/17 | 2016/2/1 | NCT00866047 | SG035-0004 | single-arm | 14894 | 14966 | 53387 | 50499 | 41090 | 31611 | NA | NA | low benefit |
| Degarelix | prostate cancer | CHN, JPN, KR | 2023/3/1 | 2012/8/28 | 2015/11/1 | NCT00295750 | FE200486 CS21 | randomized | 1521 | 1528 | 2705 | 2001 | 13928 | 1416 | NA | NA | not scorable |
| Venetoclax | acute myeloid leukemia | CHN | 2023/3/1 | 2021/3/23 | 2021/1/8 | NCT02993523 | Viale-a | randomized | 13521 | 13587 | NA | 44939 | NA | 22765 | 5.1 | no difference | high benefit |
| Carfilzomib | multiple myeloma | CHN, JPN, KR | 2023/3/1 | 2016/8/31 | 2018/2/1 | NCT01568866 | ENDEAVOR | randomized | 19139 | 19232 | 53658 | 50859 | 53152 | 44587 | 9 | no difference | low benefit |
| pralatrexate | peripheral T-cell lymphoma | JPN | NR | 2017/8/30 | NR | NCT00364923 | PDX-008 | single-arm | NA | NA | 24024 | 22910 | NA | NA | NA | NA | low benefit |
| Ramucirumab | gastric or gastro-oesophageal junction adenocarcinoma | JPN,KR | NR | 2015/5/20 | 2018/5/1 | NCT01170663 | RAINBOW | randomized | NA | NA | 31420 | 30025 | 14525 | 12852 | 2.2 | no difference | low benefit |
| Abiraterone | prostate cancer (castration-resistant) | CHN, JPN, KR | 2017/7/13 | 2014/9/2 | 2018/5/1 | NCT00638690 | COU-AA-30 | randomized | 22968 | 15455 | 28778 | 26600 | 8824 | 6237 | 4.6 | improvement | high benefit |
| Abiraterone | prostate cancer (castration-sensitive) | KR | 2020/1/1 | 2018/2/16 | 2018/5/1 | NCT01715285 | LATITUDE | randomized | NA | 49843 | NA | 85786 | 28457 | 20113 | 16.8 | improvement | high benefit |
| Lenalidomide | indolent lymphoma | no | NR | 2022/2/21 | 2020/6/18 | NCT01938001 | AUGMENT | randomized | NA | NA | NA | 61978 | NA | 23628 | 25.3 | NA | low benefit |
| Lenalidomide | multiple myeloma | no | NR | 2015/12/21 | NA | NCT00689936 | CC-5013-MM020 | randomized | NA | NA | NA | 129121 | NA | 31115 | 10.0 | improvement | high benefit |
| Lenalidomide | multiple myeloma | CHN, JPN, KR | 2017/7/13 | 2010/7/16 | 2014/4/1 | NCT00056160 | CC-5013-MM-009 | randomized | 45436 | 38207 | 93774 | 76781 | 55365 | 18502 | 9.4 | NA | high benefit |
| Everolimus | neuroendocrine tumours of the lung or gastrointestinal tract | no | 2020/1/1 | 2016/8/26 | 2016/5/27 | NCT01524783 | RADIANT 4 | randomized | NA | 9823 | NA | 43862 | NA | 6437 | 7.2 | no difference | low benefit |
| Everolimus | pancreatic neuroendocrine tumors | CHN | 2017/7/13 | 2011/12/22 | 2011/11/11 | NCT00510068 | RADIANT 3 | randomized | 13306 | 9458 | NA | 42234 | NA | 6198 | 6.3 | NA | low benefit |
| Everolimus | renal cell carcinoma | CHN, JPN, KR | 2017/7/13 | 2010/4/16 | 2011/8/1 | NCT00410124 | RECORD-1 | randomized | 6890 | 4897 | 30154 | 21869 | 12573 | 3209 | 0.4 | no difference | low benefit |
| Everolimus | subependymal giant cell astrocytomas associated with tuberous sclerosis complex | KR | 2020/1/1 | 2012/11/21 | 2011/8/1 | NCT00789828 | EXIST-1 | randomized | NA | 30531 | NA | 136333 | 78378 | 20006 | NA | NA | low benefit |
| Everolimus | angiomyolipoma | CHN | 2017/7/13 | 2012/11/21 | 2012/11/18 | NCT00790400 | EXIST-2 | randomized | 12999 | 9239 | NA | 41257 | NA | 6054 | NA | NA | low benefit |
| Everolimus | breast cancer | no | 2023/3/1 | 2014/3/17 | 2012/11/18 | NCT00863655 | BOLERO-2 | randomized | NA | 3550 | NA | 15851 | NA | 4531 | 4.4 | no difference | low benefit |
| Axitinib | renal cell carcinoma | CHN, JPN, KR | 2018/9/30 | 2012/8/28 | 2018/7/1 | NCT00678392 | AXIS | randomized | 16246 | 14361 | 37960 | 26720 | 15497 | 13733 | 0.9 | no difference | high benefit |
| Azacitidine | myelodysplastic syndromes | CHN, JPN, KR | 2018/9/30 | 2011/3/11 | 2008/4/1 | NCT00071799 | AZA PH GL 2003 CL 001 | randomized | 21204 | 17758 | 53270 | 37129 | 41035 | 15123 | 9.5 | NA | high benefit |
| Azacitidine | acute myeloid leukemia | CHN, KR | 2018/9/30 | 2021/3/23 | 2008/4/1 | NCT01074047 | AZA-AML-001 | randomized | 14136 | 11839 | NA | 24753 | 27356 | 10082 | 3.9 | no difference | low benefit |
| Crizotinib | NSCLC （ALK-positive） | CHN, JPN, KR | 2018/9/30 | 2012/5/29 | 2015/5/1 | NCT01154140 | PROFILE 1014 | randomized | 36580 | 22466 | 86936 | 78140 | 99171 | 35607 | 3.9 | improvement | high benefit |
| Crizotinib | NSCLC （ROS1-positive） | CHN | 2018/9/30 | 2017/5/18 | 2017/9/21 | CTR20140093 | A8081063 | single-arm | 45787 | 28120 | NA | 97807 | NA | 44570 | NA | NA | low benefit |
| Pazopanib | renal cell carcinoma | CHN, KR | 2018/9/30 | 2014/3/17 | 2011/6/1 | NCT00334282 | VEG105192 | randomized | 22664 | 21090 | NA | 28339 | 24284 | 18551 | 2.4 | no difference | low benefit |
| Regorafenib | colorectal cancer | CHN, JPN, KR | 2018/9/30 | 2013/5/24 | 2016/6/1 | NCT01103323. | CORRECT | randomized | 7879 | 6452 | 11560 | 10890 | 9529 | 6609 | 1.4 | no difference | low benefit |
| Regorafenib | gastrointestinal stromal tumours | CHN | 2018/9/30 | 2013/8/20 | 2013/11/13 | NCT01271712 | GRID | randomized | 15035 | 12312 | NA | 20782 | NA | 12612 | 3.9 | no difference | low benefit |
| Regorafenib | hepatocellular carcinoma | CHN | 2018/9/30 | 2017/6/26 | 2017/7/12 | NCT01774344. | RESORCE | randomized | 21948 | 17973 | NA | 30336 | NA | 18410 | 2.8 | no difference | high benefit |
| Vemurafenib | melanoma | CHN, JPN, KR | 2018/9/30 | 2015/2/24 | 2017/7/1 | NCT01006980 | BRIM-3 | randomized | 28299 | 21088 | 64082 | 60553 | 38022 | 33362 | 3.9 | NA | high benefit |
| Ibrutinib | mantle-cell lymphoma | CHN | 2018/9/30 | 2016/12/2 | NA | NCT01646021 | PCI-32765MCL3001 | randomized | 52096 | 43347 | NA | 136790 | NA | 78349 | 6.8 | NA | high benefit |
| Ibrutinib | chronic lymphocytic leukemia or small lymphocytic lymphoma | CHN, JPN, KR | 2018/9/30 | 2016/5/25 | 2016/6/1 | NCT01578707 | PCYC-1112-CAM | randomized | 111247 | 92565 | 281586 | 292104 | 216701 | 167307 | 2.6 | improvement | high benefit |
| Ibrutinib | Waldenström’s macroglobulinemia(monotherapy) | no | 2021/3/1 | 2022/11/30 | NA | NCT01614821 | PCYC-1118E-CA | single-arm | NA | 43122 | NA | 136078 | NA | 77941 | NA | NA | low benefit |
| Ibrutinib | Waldenström’s macroglobulinemia (plus Rituximab) | no | 2021/3/1 | 2022/11/30 | NA | NCT02165397 | PCYC-1127-CA | randomized | NA | 58248 | NA | 183812 | NA | 105281 | NA | no difference | not scorable |
| Ixazomib | multiple myeloma | CHN, JPN, KR | 2018/9/30 | 2017/5/24 | 2021/3/1 | NCT01564537 | C16010 | randomized | 42492 | 39540 | 70491 | 67292 | 66154 | 60630 | 2.0 | no difference | low benefit |
| Olaparib | ovarian cancer and a BRCA mutation | KR | 2021/3/1 | 2019/6/18 | 2021/10/1 | NCT01844986 | SOLO1 | randomized | NA | 39331 | NA | 116400 | 119327 | 110983 | 42.2 | no difference | high benefit |
| Olaparib | ovarian cancer and a BRCA1/2 mutation | CHN, JPN, KR | 2020/1/1 | 2018/4/18 | 2021/10/1 | NCT01874353 | SOLO2 | randomized | 53694 | 46525 | 167238 | 137693 | 141155 | 131285 | 12.9 | no difference | high benefit |
| Olaparib | prostate cancer | no | 2023/3/1 | 2020/12/25 | 2021/10/6 | NCT02987543. | PROfound | randomized | NA | 12151 | NA | 35961 | NA | 34287 | 4.4 | improvement | low benefit |
| Pertuzumab | breast cancer (adjuvant therapy) | CHN | 2020/1/1 | 2018/10/10 | 2018/4/11 | NCT01358877 | APHINITY | randomized | 14192 | 13974 | NA | 29833 | NA | 32582 | NA | no difference | high benefit |
| Pertuzumab | breast cancer (neoadjuvant therapy) | CHN, KR | 2020/1/1 | 2018/10/10 | 2017/6/1 | NCT00545688 | NEOSPHERE | randomized | 3735 | 3677 | NA | 7851 | 12287 | 8574 | NA | NA | low benefit |
| Pertuzumab | breast cancer (metastatic) | JPN,KR | NR | 2013/8/27 | 2017/6/1 | NCT00567190 | CLEOPATRA | randomized | NA | NA | 17555 | 14131 | 22117 | 15433 | 16.3 | no difference | high benefit |
| Enzalutamide | prostate cancer (nonmetastatic castration-resistant) | JPN | 2023/3/1 | 2014/5/23 | 2019/2/7 | NCT02003924 | PROSPER | randomized | NA | 42027 | 104129 | 73977 | NA | 65777 | 10.7 | no difference | low benefit |
| Enzalutamide | prostate cancer (metastatic castration-resistant) | CHN | 2021/3/1 | 2020/5/29 | 2021/9/14 | NCT01212991 | PREVAIL | randomized | 22943 | 22563 | NA | 39716 | NA | 35314 | 4.0 | improvement | high benefit |
| Lenvatinib | hepatocellular carcinoma | CHN | 2021/3/1 | 2018/3/23 | 2018/8/29 | NCT01761266 | REFLECT | randomized | 8362 | 8224 | NA | 15704 | NA | 11824 | 1.3 | no difference | low benefit |
| Lenvatinib | thyroid cancer | JPN | 2023/3/1 | 2015/5/19 | 2015/10/7 | NCT01321554 | SELECT | randomized | NA | 33405 | 76056 | 72603 | NA | 28625 | 14.7 | NA | low benefit |
| Niraparib | ovarian cancer （platinum-sensitive） | CHN, JPN, KR | 2021/3/1 | 2020/11/18 | 2019/11/1 | NCT01847274 | NOVA | randomized | 57052 | 42867 | 157588 | 149049 | 124891 | 102052 | 15.5 | no difference | low benefit |
| Niraparib | advanced ovarian cancer | no | 2022/1/1 | 2020/9/25 | 2019/12/24 | NCT02655016 | PRIMA | randomized | NA | 48990 | NA | 170342 | NA | 116631 | 6.6 | no difference | low benefit |
| Trametinib | melanoma (adjuvant therapy) | CHN | 2021/3/1 | 2018/7/2 | 2019/5/14 | NCT01682083 | COMBI-AD | randomized | 18404 | 16289 | NA | 74178 | NA | 32615 | NA | NA | high benefit |
| Trametinib | melanoma | CHN, JPN, KR | 2021/3/1 | 2016/5/25 | 2017/11/1 | NCT01584648 | COMBI-D | randomized | 33462 | 29617 | 141856 | 134869 | 88396 | 59300 | 6.4 | improvement | high benefit |
| Trametinib | NSCLC | no | 2023/3/1 | 2018/3/23 | 2018/3/12 | NCT01336634 | CDRB436E2201 | single-arm | NA | 15697 | NA | 71481 | NA | 31429 | NA | NA | low benefit |
| Abemaciclib | breast cancer (plus either anastrozole or letrozole) | CHN, JPN, KR | 2022/1/1 | 2018/11/20 | 2020/6/1 | NCT02246621 | MONARCH 3 | randomized | 13530 | 13424 | 72013 | 69722 | 45529 | 40565 | 13.4 | no difference | low benefit |
| Abemaciclib | breast cancer (plus Fulvestrant ) | CHN, JPN, KR | 2022/1/1 | 2018/11/20 | 2020/6/1 | NCT02107703 | MONARCH 2 | randomized | 13459 | 13354 | 71634 | 69355 | 45289 | 40351 | 9.4 | no difference | high benefit |
| Abemaciclib | breast cancer (adjuvant therapy) | no | NR | 2021/12/24 | 2022/11/18 | NCT03155997 | monarchE | randomized | NA | NA | NA | 95671 | NA | 55662 | NA | no difference | high benefit |
| Apalutamide | prostate cancer (metastasis-free) | CHN, JPN | 2022/1/1 | 2019/5/22 | 2023/4/10 | NCT01946204 | SPARTAN | randomized | 39988 | 39676 | 88722 | 84639 | NA | 75434 | 14.0 | no difference | high benefit |
| Apalutamide | prostate cancer (metastatic castration-sensitive) | CHN, KR | 2022/1/1 | 2020/5/29 | 2023/4/1 | NCT02489318. | TITAN | randomized | 38803 | 38501 | NA | 82131 | 73410 | 73199 | NA | no difference | high benefit |
| Eribulin | breast cancer | CHN, JPN, KR | 2022/1/1 | 2011/7/19 | 2014/4/1 | NCT00388726 | EMBRACE | randomized | 3631 | 3602 | 18112 | 16996 | 5678 | 4541 | 2.7 | NA | low benefit |
| Obinutuzumab | lymphoma | CHN, JPN | 2022/1/1 | 2018/8/29 | 2017/12/1 | NCT01332968 | GALLIUM | randomized | 11213 | 11125 | 28887 | 27912 | NA | 24761 | NA | no difference | low benefit |
| Dacomitinib | NSCLC | CHN, JPN, KR | 2022/1/1 | 2019/2/26 | 2020/12/1 | NCT01774721 | ARCHER 1050 | randomized | 2146 | 2129 | 14275 | 12197 | 6360 | 5742 | 7.1 | reduction | low benefit |
| Daratumumab | multiple myeloma (newly diagnosed) | CHN | 2022/1/1 | 2019/8/22 | 2020/1/21 | NCT02252172 | MMY3008 | randomized | 128397 | 127396 | NA | 256833 | NA | 72532 | NA | improvement | not scorable |
| Daratumumab | multiple myeloma (previously treated ) | CHN | 2022/1/1 | 2019/8/22 | 2019/8/21 | NCT02136134 | MMY3004 | randomized | 46833 | 46468 | NA | 95416 | NA | 79369 | 9.6 | no difference | low benefit |
| Daratumumab | multiple myeloma (monotherapy) | CHN, JPN, KR | 2022/1/1 | 2017/11/22 | 2019/5/1 | NCT01985126 | MMY2002 | single-arm | 25258 | 25062 | 53634 | 51460 | 48696 | 42806 | NA | NA | low benefit |
| Darolutamide | prostate cancer | CHN, JPN | 2022/1/1 | 2020/4/22 | NR | NCT02200614 | ARAMIS | randomized | 16632 | 16503 | 32770 | 31103 | NA | NA | 22.0 | improvement | low benefit |
| Blinatumomab | acute lymphoblastic leukemia | JPN | NR | 2018/11/20 | 2018/1/30 | NCT02013167 | TOWER | randomized | NA | NA | 114790 | 110907 | NA | 76396 | 3.7 | improvement | high benefit |
| Durvalumab | NSCLC | JPN,KR | NR | 2018/8/29 | 2020/4/1 | NCT02125461 | PACIFIC | randomized | NA | NA | 109761 | 93865 | 85773 | 76714 | 18.4 | no difference | high benefit |
| Nivolumab | NSCLC | no | NR | 2015/12/17 | 2017/9/1 | NCT01642004 | CheckMate 017 | randomized | NA | NA | NA | 22291 | NA | 18474 | 3.2 | NA | high benefit |
| Nivolumab | squamous-cell carcinoma of the head and neck | no | NR | 2017/3/24 | 2018/3/23 | NCT02105636 | CHECKMATE-141 | randomized | NA | NA | NA | 11146 | NA | 9237 | 2.4 | improvement | high benefit |
| Nivolumab | gastric, gastro-oesophageal junction, and oesophageal adenocarcinoma | no | NR | 2020/2/21 | 2021/6/10 | NCT02872116 | CheckMate 649 | randomized | NA | NA | NA | 41796 | NA | 34638 | 3.3 | no difference | high benefit |
| Nivolumab | gastric or gastro-oesophageal junction cancer | no | NR | 2017/9/22 | 2018/10/5 | [NCT02267343](https://clinicaltrials.gov/show/NCT02267343) | CA209316 | randomized | NA | NA | NA | 11146 | NA | 9237 | 1.1 | NA | low benefit |
| Nivolumab | pleural mesothelioma | no | NR | 2018/9/21 | 2021/6/10 | NCT02899299 | CheckMate 649 | randomized | NA | NA | NA | 22291 | NA | 18474 | 4.0 | NA | low benefit |
| Nivolumab | esophageal or gastroesophageal junction cancer | no | NR | 2021/11/25 | 2022/2/14 | NCT02743494 | CheckMate 577 | randomized | NA | NA | NA | 61301 | NA | 50803 | NA | no difference | high benefit |
| Nivolumab | esophageal squamous-cell carcinoma | no | NR | 2020/8/21 | 2023/3/22 | NCT03143153 | CheckMate 648 | randomized | NA | NA | NA | 33437 | NA | 27711 | 2.5 | no difference | low benefit |
| Pembrolizumab | melanoma | JPN,KR | NR | 2017/2/15 | 2017/9/1 | NCT01704287 | KEYNOTE-002 | randomized | NA | NA | 33480 | 16312 | 25070 | 16320 | 2.4 | improvement | low benefit |
| Pembrolizumab | nonsquamous NSCLC | no | NR | 2018/12/21 | 2019/5/13 | NCT02578680 | KEYNOTE-189 | randomized | NA | NA | NA | 45673 | NA | 45696 | 11.3 | improvement | high benefit |
| Pembrolizumab | squamous NSCLC | no | NR | 2018/12/21 | 2019/9/30 | NCT02775435 | KEYNOTE-407 | randomized | NA | NA | NA | 32624 | NA | 32640 | 5.5 | improvement | high benefit |
| Pembrolizumab | NSCLC | KR | NR | 2016/12/19 | 2017/9/1 | NCT02220894 | KEYNOTE-042 | randomized | NA | NA | NA | 29361 | 45126 | 29376 | 7.8 | NA | high benefit |
| Pembrolizumab | esophageal cancer | no | NR | 2020/8/21 | 2022/3/7 | NCT02564263 | KEYNOTE-181 | randomized | NA | NA | NA | 19574 | NA | 19584 | 3.6 | no difference | high benefit |
| Pembrolizumab | oesophageal cancer | no | NR | 2021/11/25 | 2022/3/7 | NCT03189719 | KEYNOTE-590 | randomized | NA | NA | NA | 55460 | NA | 55488 | 2.6 | no difference | low benefit |
| Pembrolizumab | squamous-cell carcinoma of the head and neck | no | NR | 2019/12/20 | 2020/8/27 | NCT02358031 | KEYNOTE-048 | randomized | NA | NA | NA | 16312 | NA | 16320 | 4.2 | no difference | high benefit |
| Pembrolizumab | colorectal cancer | no | NR | 2021/8/25 | 2022/3/7 | NCT02563002 | KEYNOTE-177 | randomized | NA | NA | NA | 52198 | NA | 52224 | 8.3 | improvement | high benefit |
| Palbociclib | breast cancer | JPN,KR | NR | 2017/11/22 | 2017/11/1 | NCT01740427 | PALOMA-2 | randomized | NA | NA | 87584 | 84033 | 60052 | 42187 | 13.1 | no difference | low benefit |
| Atezolizumab | NSCLC (Adjuvant therapy) | no | NR | 2022/5/26 | 2022/9/27 | NCT02486718 | IMpower010 | randomized | NA | NA | NA | 68615 | NA | 28137 | NA | NA | high benefit |
| Atezolizumab | hepatocellular carcinoma | no | NR | 2020/9/25 | NA | NCT03434379 | IMbrave150 | randomized | NA | NA | NA | 47172 | NA | 19344 | 5.8 | improvement | high benefit |
| Atezolizumab | small-cell lung cancer | no | NR | 2019/8/22 | NA | NCT02763579 | IMpower133 | randomized | NA | NA | NA | 30019 | NA | 12310 | 2.0 | improvement | low benefit |
| Atezolizumab | NSCLC （first-line） | no | NR | 2019/9/20 | NA | NCT02409342 | IMpower110 | randomized | NA | NA | NA | 34307 | NA | 14069 | 7.1 | NA | high benefit |
| Atezolizumab | nonsquamous NSCLC | JPN | NR | 2018/4/18 | 2018/3/14 | NCT02657434 | IMpower132 | randomized | NA | NA | 45451 | 38596 | NA | 15827 | 4.5 | NA | high benefit |
| Pemigatinib | cholangiocarcinoma | JPN | NR | 2021/5/19 | NR | NCT02924376 | FIGHT-202 | single-arm | NA | NA | 86642 | 81865 | NA | NA | NA | NA | low benefit |
| Bendamustine | Hodgkin's lymphoma | CHN, JPN, KR | 2022/1/1 | 2010/12/10 | 2018/9/1 | NA | no common trial | NA | 3896 | 2800 | 18765 | 16823 | 5847 | NA | NA | NA | NA |
| Inotuzumab ozogamicin | acute lymphoblastic leukemia | JPN,KR | NR | 2018/4/18 | 2019/10/1 | NCT01564784; | INO-VATE ALL | randomized | NA | NA | 73863 | 70869 | 71188 | 62039 | 1.5 | NA | high benefit |

Notes: NSCLC, non-small cell lung cancer. NR:not reimbursed. NA:not available or not applicable.

### **Data availability for clinical value of the 91 included indications**

**Survival (OS, or PFS when OS was not available)**

Of these 91 indications, ten were supported by single-arm clinical trials and were, therefore, not included in the analysis of the relationship between treatment price and survival. For the remaining 81 indications supported by randomized controlled trials, 55 had available overall survival (OS) data, and 11 had progression-free survival (PFS) data available as a surrogate, leaving 15 indications without available OS or PFS data. Among these 15 indications, four did not have OS as a clinical endpoint, while the OS median times for the remaining indications were reported as not mature, not reached, not estimable, or not reported.

**Quality of life**

Of these 91 indications, ten were supported by single-arm clinical trials and were, therefore, not included in the analysis of the relationship between treatment price and quality of life (QoL). For the remaining 81 indications supported by randomized controlled trials, 38 showed no difference in QoL compared to their reference drugs, 20 showed improvement, and 1 showed reduction, leaving 22 indications with QoL data not available. In such cases, we categorized QoL into three groups: improvement (N=20), no difference (N=38), and reduced or unavailable (N=23).

**ESMO-MCBS scores**

Since ESMO-MCBS can assess the clinical value of single-arm clinical trials, we included them in the analysis of the relationship between treatment price and ESMO-MCBS scores. Of these 91 indications, 87 had available ESMO scores from the ESMO website (solid tumors [60]), published work of the ESMO working group (hematological malignancies [n=9]), or were assessed by authors using ESMO-MCBS evaluation forms after studying the online tutorials (including both solid tumors [9] and hematological malignancies [n=10]). The remaining four indications could not be assessed, including three indications that were not scorable using ESMO-MCBS evaluation forms, and one indication did not have a common clinical trial across the three countries for assessment. For the 87 indications with available ESMO scores, 45 were categorized as high benefit for having a score of A-B in the curative setting or 4-5 in the non-curative setting, and 42 were categorized as low benefit for having any other scores.

Of the 22 indications for which ESMO-MCBS was assessed by authors, disagreements occurred for three indications, and consensus was reached through discussion.

### **Data availability for each analysis**

**Table S2. Explanation for price-value analyses of listed indications as of June 2023**

| **Price-survival analysis** | **Sample size (n)** | **Explanation for missing data** |
| --- | --- | --- |
| China (N=60) | 40 (OS=32, PFS=8) | Single-arm clinical trials=8  No OS endpoint and PFS not available =4  OS and PFS not available=8 |
| Japan (N=91) | 66 (OS=55, PFS=11) | Single-arm clinical trials=10  No OS endpoint and PFS not available=4  OS and PFS not available=11 |
| South Korea (N=87) | 65 (OS=55, PFS=10) | Single-arm clinical trials=10  No OS endpoint and PFS not available=4  OS and PFS not available=11 |
| **Price-QoL analysis** | **Sample size (n)** | **Explanation for missing data*** |
| China (N=60) | 52  reduced or unavailable=14  no difference=27  improvement=11 | Single-arm clinical trials=8  QoL not available=13 |
| Japan (N=91) | 81  reduced or unavailable=23  no difference=38  improvement=20 | Single-arm clinical trials=10  QoL not available=22 |
| South Korea (N=87) | 80  reduced or unavailable=23  no difference=38  improvement=19 | Single-arm clinical trials=7  QoL not available=22 |
| **Price-ESMO-MCBS analysis** | **Sample size (n)** | **Explanation for missing data** |
| China (N=60) | 56  low benefit=31  high benefit=25 | not scorable=3  no common trial for assessment=1 |
| Japan (N=91) | 87  low benefit=42  high benefit=45 | not scorable=3  no common trial for assessment=1 |
| South Korea (N=87) | 84  low benefit=39  high benefit=45 | not scorable=3 |

Notes: For indications with unavailable QoL, we categorized them as reduced or unavailable. Therefore, these indications were included for analyses.

**Table S3. Explanation for price-value analyses of initial listed indications using latest prices and clinical value**

| **Price-survival analysis** | **Sample size (N)** | **Explanation for missing data** |
| --- | --- | --- |
| China (N=48) | 32 (OS=26, PFS=6) | Single-arm clinical trials=6  No OS endpoint and PFS not available =3  OS and PFS not available=7 |
| Japan (N=41) | 32 (OS=26, PFS=6) | Single-arm clinical trials=7  No OS endpoint and PFS not available =1  OS and PFS not available=1 |
| South Korea (N=41) | 31 (OS=26, PFS=5) | Single-arm clinical trials=5  No OS endpoint and PFS not available=3  OS and PFS not available=2 |
| **Price-QoL analysis** | **Sample size (n)** | **Explanation for missing data*** |
| China (N=48) | 42  reduced or unavailable=12  no difference=21  improvement=9 | Single-arm clinical trials=6  QoL not available=11 |
| Japan (N=41) | 34  reduced or unavailable=9  no difference=16  improvement=9 | Single-arm clinical trials=7  QoL not available=8 |
| South Korea (N=41) | 36  reduced or unavailable=10  no difference=18  improvement=8 | Single-arm clinical trials=5  QoL not available=9 |
| **Price-ESMO-MCBS analysis** | **Sample size (n)** | **Explanation for missing data** |
| China (N=48) | 45  low benefit=22  high benefit=23 | not scorable=2  no common trial for assessment=1 |
| Japan (N=41) | 39  low benefit=20  high benefit=19 | not scorable=3  no common trial for assessment=1 |
| South Korea (N=41) | 39  low benefit=18  high benefit=21 | not scorable=1  no common trial for assessment=1 |

Notes: For indications with unavailable QoL, we categorized them as reduced or unavailable. Therefore, these indications were included for analyses.

**Table S4. Explanation for price-value analyses of initial listed indications using initial prices and initial clinical value**

| **Price-survival analysis** | **Sample size (N)** | **Explanation for missing data** |
| --- | --- | --- |
| China (N=48) | 32 (OS=25, PFS=7) | Single-arm clinical trials=6  No OS endpoint and PFS not available =3  OS and PFS not available=7 |
| Japan (N=41) | 30 (OS=18, PFS=12) | Single-arm clinical trials=7  No OS endpoint and PFS not available =1  OS and PFS not available=3 |
| South Korea (N=41) | 28 (OS=16, PFS=12) | Single-arm clinical trials=5  No OS endpoint and PFS not available=3  OS and PFS not available=5 |
| **Price-QoL analysis** | **Sample size (n)** | **Explanation for missing data*** |
| China (N=48) | 42  reduced or unavailable=13  no difference=20  improvement=9 | Single-arm clinical trials=6  QoL not available=12 |
| Japan (N=41) | 34  reduced or unavailable=14  no difference=11  improvement=9 | Single-arm clinical trials=7  QoL not available=13 |
| South Korea (N=41) | 36  unavailable=15  no difference=14  improvement=7 | Single-arm clinical trials=5  QoL not available=15 |
| **Price-ESMO-MCBS analysis** | **Sample size (n)** | **Explanation for missing data** |
| China (N=48) | 45  low benefit=23  high benefit=22 | not scorable=2  no common trial for assessment=1 |
| Japan (N=41) | 37  low benefit=21  high benefit=16 | not scorable=3  no common trial for assessment=1 |
| South Korea (N=41) | 36  low benefit=17  high benefit=19 | not scorable=4  no common trial for assessment=1 |

Notes: For indications with unavailable QoL, we categorized them as reduced or unavailable. Therefore, these indications were included for analyses.

### **Correlations of initial and latest treatment prices with clinical value for initial listed indications**

### **Table S5. Correlations of initial and latest treatment prices with clinical value for initial listed indications**

| **Clinical value** | **Initial treatment prices and initial clinical value** | | | **Latest treatment prices and latest clinical value** | | |
| --- | --- | --- | --- | --- | --- | --- |
|  | **China** | **Japan** | **South Korea** | **China** | **Japan** | **South Korea** |
| Survival | *r*=0·35 (0·001, 0·62)  p=0·050, N=32 | *r*=0·38 (0·023, 0·65)  p=0·038, N=30 | *r*=0·35 (-0·038, 0·64)  p=0·076, N=28 | *r*=0·37 (0·030, 0·64)  p=0·035, N=32 | *r*=0·36 (0·011, 0·63)  p=0·044, N=32 | *r*=0·42 (0·082, 0·68)  p=0·017, N=31 |
| QoL | *r*=0·41 (0·12, 0·63)  p=0·0072, N=42 | *r*=0·15 (-0·20, 0·46)  p=0·41, N=34 | *r*=0·24 (-0·10, 0·53)  p=0·16, N=36 | *r*=0·39 (0·10, 0·62)  p=0·011, N=42 | *r*=0·28(-0·060,0·57)  p=0·10, N=34 | *r*=0·33 (0·007, 0·60)  p=0·046, N=36 |
| ESMO-MCBS | *r*=0·37 (0·089, 0·60)  p=0·012, N=45 | *r*=0·28 (-0·047, 0·55)  p=0·092, N=37 | *r*=0·40 (0·087, 0·65)  p=0·014, N=36 | *r*=0·43 (0·15, 0·64)  p=0·0033, N=45 | *r*=0·36 (0·050, 0·61)  p=0·024, N=39 | *r*=0·36 (0·051, 0·61)  p=0·024, N=39 |

Notes: For each analysis, we excluded indications with missing required data on clinical value.

### **Robust analysis using OS as the measure of survival instead of using aggregated survival in either OS or PFS**

### **Table S6. Comparison of correlations between treatment prices and survival when using aggregated survival (either OS or PFS) versus using only OS for initial listed indications.**

| **Initial clinical value** | **Initial treatment prices** | | |
| --- | --- | --- | --- |
|  | **China** | **Japan** | **South Korea** |
| Survival | *r*=0·35 (0·001, 0·62)  p=0·050, N=32 | *r*=0·38 (0·023, 0·65)  p=0·038, N=30 | *r*=0·35 (-0·038, 0·64)  p=0·076, N=28 |
| OS | *r*=0·32 (-0·089, 0·63)  p=0·12, N=25 | *r*=0·27 (-0·22, 0·66)  p=0·27, N=18 | *r*=0·21 (-0·32, 0·64)  p=0·44, N=16 |

Notes: For each analysis, we excluded indications with missing required data on clinical value.

### **Table S7. Comparison of correlations between treatment prices and survival when using aggregated survival (either OS or PFS) versus using only OS for listed indications as of June 2023.**

| **Latest clinical Value** | **Latest treatment prices** | | |
| --- | --- | --- | --- |
|  | **China** | **Japan** | **South Korea** |
| Survival | *r*=0·44 (0·15, 0·66)  p=0·0048, N=40 | *r*=0·53 (0·32, 0·68)  p<0·0001, N=66 | *r*=0·42 (0·19, 0·60)  p<0·0001, N=65 |
| OS | *r*=0·42 (0·083, 0·67)  p=0·017, N=32 | *r*=0·48 (0·25, 0·66)  p=0·00020, N=55 | *r*=0·34 (0·083, 0·56)  p=0·011, N=55 |

Notes: For each analysis, we excluded indications with missing required data on clinical value.

### **Robust analysis** **after** **restricting the sample to indications that were listed in all three countries**

**Treatment prices and clinical value**

### **Table S8. Correlations between initial treatment prices and clinical value for initial indications listed in all three countries (N=26).**

| **Initial clinical value** | **Initial treatment prices** | | |
| --- | --- | --- | --- |
|  | **China** | **Japan** | **South Korea** |
| Survival (N=20) | *r*=0·32 (-0·15, 0·67)  p=0·17 | *r*=0·47 (0·022, 0·76)  p=0·042 | *r*=0·37 (-0·12, 0·71)  p=0·13 |
| QoL (N=21) | *r*=0·41 (-0·031, 0·71)  p=0·068 | *r*=0·42 (-0·018, 0·72)  p=0·060 | *r*=0·45 (0·019, 0·74)  p=0·042 |
| ESMO-MCBS (N=24) | *r*=0·44 (0·046, 0·72)  p=0·031 | *r*=0·34 (-0·083, 0·66)  p=0·11 | *r*=0·38 (-0·038, 0·69)  p=0·073 |

Notes: For each analysis, we excluded indications with missing required data on clinical value.

### **Table S9. Correlations between latest treatment prices and clinical value for indications as of June 2023 listed in all three countries (N=58).**

| **Latest clinical value** | **Latest treatment prices** | | |
| --- | --- | --- | --- |
|  | **China** | **Japan** | **South Korea** |
| Survival (N=39) | *r*=0·46 (0·17, 0·68)  p=0·0030 | *r*=0·60 (0·34, 0·77)  p<0·0001 | *r*=0·46 (0·17, 0·68)  p=0·0030 |
| QoL (N=51) | *r*=0·32 (0·05, 0·55)  p=0·022 | *r*=0·23 (-0·044, 0·48)  p=0·097 | *r*=0·35 (0·085, 0·57)  p=0·011 |
| ESMO-MCBS (N=55) | *r*=0·34 (0·085, 0·56)  p=0·010 | *r*=0·31 (0·044, 0·53)  p=0·023 | *r*=0·30 (0·034, 0·52)  p=0·028 |

Notes: For each analysis, we excluded indications with missing required data on clinical value.

### **Robust analysis using generalized estimating equations (GEE)**

We employed GEE to examine the relationships between treatment prices and clinical value within each country, as well as across countries. Specifically, GEE was used to estimate the correlations between both initial and latest treatment prices and clinical value (survival, quality of life, or ESMO-MCBS scores) within each country. This corresponds to the Spearman rank correlation coefficients employed in the main analyses. Additionally, GEE was utilized to investigate whether the association between each measure of clinical value and the percentage changes in treatment prices was modified by the country setting. This corresponds to the multiple linear regression models employed in the main analyses. The parameters of GEE remained the same as those in the main analyses and the independence structure was used in GEE.

### **Table S10. Associations of initial treatment prices with initial clinical value for initial listed indications in China, Japan, and South Korea.**

| **Variables** | **log (initial treatment prices)** | | | | | |
| --- | --- | --- | --- | --- | --- | --- |
|  | **China** | | **Japan** | | **South Korea** | |
|  | **Coefficient (95% CI)** | **P value** | **Coefficient (95% CI)** | **P value** | **Coefficient (95% CI)** | **P value** |
| Survival | 0·018 (0·000, 0·035) | 0·045 | 0·015 (-0·005, 0·035) | 0·13 | 0·033 (0·000, 0·065) | 0·048 |
| QoL_no difference | 0·277 (-0·014, 0·567) | 0·062 | 0·005(-0·298, 0·309) | 0·97 | 0·058 (-0·241, 0·357) | 0·70 |
| QoL_improvement | 0·515 (0·210, 0·820) | 0·00095 | 0·218 (-0·089, 0·527) | 0·16 | 0·253 (-0·158, 0·664) | 0·23 |
| ESMO_high benefit | 0·263 (0·084, 0·441) | 0·0039 | 0·194 (0·000, 0·387) | 0·050 | 0·308 (0·063, 0·553) | 0·014 |

Notes: For each analysis, we excluded indications with missing required data on clinical value. Reference categories: QoL: reduction or NA; ESMO: low benefit.

### **Table S11. Associations of latest treatment prices with latest clinical value for listed indications as of June 2023 in China, Japan, and South Korea.**

| **Variables** | **log (latest treatment prices)** | | | | | |
| --- | --- | --- | --- | --- | --- | --- |
|  | **China** | | **Japan** | | **South Korea** | |
|  | **Coefficient (95% CI)** | **P value** | **Coefficient (95% CI)** | **P value** | **Coefficient (95% CI)** | **P value** |
| Survival | 0·018 (0·005, 0·030) | 0·0048 | 0·019 (0·009, 0·030) | 0·00023 | 0·023 (0·012, 0·034) | <0·0001 |
| QoL_no difference | 0·214 (-0·089, 0·518) | 0·17 | 0·114 (-0·115, 0·344) | 0·33 | 0·269 (0·058, 0·479) | 0·013 |
| QoL_improvement | 0·414 (0·136, 0·692) | 0·0035 | 0·210 (0·004, 0·417) | 0·046 | 0·310 (0·098, 0·522) | 0·0041 |
| ESMO_high benefit | 0·249 (0·083, 0·415) | 0·0033 | 0·140 (0·022, 0·258) | 0·020 | 0·222 (0·011, 0·434) | 0·039 |

Notes: For each analysis, we excluded indications with missing required data on clinical value. Reference categories: QoL: reduction or NA; ESMO: low benefit.

### **Table S12. Regression analyses for initial treatment prices and clinical value in China, Japan, and South Korea for initial listed indications.**

| **Variables** | **log (initial treatment prices)** | | | | | |
| --- | --- | --- | --- | --- | --- | --- |
|  | **Model (1)** | | **Model (2)** | | **Model (3)** | |
|  | **Coefficient (95% CI)** | **P value** | **Coefficient (95% CI)** | **P value** | **Coefficient (95% CI)** | **P value** |
| Survival | 0·018 (0·000, 0·035) | 0·045 |  |  |  |  |
| QoL_no difference |  |  | 0·277 (-0·014, 0·567) | 0·062 |  |  |
| QoL_improvement |  |  | 0·515 (0·210, 0·820) | 0.00095 |  |  |
| ESMO_high benefit |  |  |  |  | 0·262 (0·084, 0·441) | 0·0039 |
| Japan | 0·480 (0·195, 0·765) | 0.00095 | 0·630 (0·290, 0·971) | 0.00028 | 0·514 (0·313· 0·715) | <0·0001 |
| South Korea | 0·170 (-0·161, 0·501) | 0·31 | 0·471 (0·153, 0·789) | 0·0037 | 0·264 (0·024, 0·504) | 0·031 |
| Survival # Japan | -0·002 (-0·029, 0·024) | 0·86 |  |  |  |  |
| Survival # South Korea | 0·015 (-0·022, 0·052) | 0·43 |  |  |  |  |
| QoL_no difference # Japan |  |  | -0·272 (-0·692, 0·149) | 0·21 |  |  |
| QoL_improvement # Japan |  |  | -0·297 (-0·730, 0·137) | 0·18 |  |  |
| QoL_no difference # South Korea |  |  | -0·219 (-0·635, 0·198) | 0·30 |  |  |
| QoL_improvement # South Korea |  |  | -0·262 (-0·774, 0·250) | 0·32 |  |  |
| ESMO_high benefit # Japan |  |  |  |  | -0·069 (-0·332, 0·194) | 0·61 |
| ESMO_high benefit # South Korea |  |  |  |  | 0·045 (-0·257, 0·348) | 0·77 |

Notes: For each analysis, we excluded indications with missing required data on clinical value. Treatment prices were calculated based on initial prices. Survival: the absolute differences in median overall survival or progression-free survival between the experimental and control groups. QoL=quality of life, NA=not available. Reference categories: QoL: reduction or NA; ESMO: low benefit; country: China.

### **Table S13. Regression analyses for latest treatment prices and clinical value in China, Japan, and South Korea for listed indications as of June 2023.**

| **Variables** | **log (latest treatment prices)** | | | | | |
| --- | --- | --- | --- | --- | --- | --- |
|  | **Model (1)** | | **Model (2)** | | **Model (3)** | |
|  | **Coefficient (95% CI)** | **P value** | **Coefficient (95% CI)** | **P value** | **Coefficient (95% CI)** | **P value** |
| Survival | 0·018 (0·005, 0·030) | 0·0048 |  |  |  |  |
| QoL_no difference |  |  | 0·214 (-0·089, 0·518) | 0·17 |  |  |
| QoL_improvement |  |  | 0·414 (0·136, 0·692) | 0·0035 |  |  |
| ESMO_high benefit |  |  |  |  | 0·249 (0·083, 0·415) | 0·0033 |
| Japan | 0·371 (0·128, 0·614) | 0·0027 | 0·488 (0·186, 0·790) | 0·0015 | 0·440 (0·254, 0·625) | <0·0001 |
| South Korea | 0·111 (-0·150, 0·373) | 0·41 | 0·151 (-0·137, 0·439) | 0·30 | 0·155(-0·093, 0·404) | 0·22 |
| Survival # Japan | 0·002 (-0·015, 0·018) | 0·85 |  |  |  |  |
| Survival # South Korea | 0·005 (-0·011, 0·022) | 0·52 |  |  |  |  |
| QoL_no difference # Japan |  |  | -0·100 (-0·480, 0·281) | 0·61 |  |  |
| QoL_improvement # Japan |  |  | -0·204 (-0·550, 0·142) | 0·25 |  |  |
| QoL_no difference # South Korea |  |  | 0·054 (-0·315, 0·424) | 0·77 |  |  |
| QoL_improvement # South Korea |  |  | -0·104 (-0·454, 0·245) | 0·56 |  |  |
| ESMO_high benefit # Japan |  |  |  |  | -0·109 (-0·313, 0·094) | 0·29 |
| ESMO_high benefit # South Korea |  |  |  |  | -0·026 (-0·295, 0·242) | 0·85 |

Notes: For each analysis, we excluded indications with missing required data on clinical value. Treatment prices were calculated based on latest prices. Survival: the absolute differences in median overall survival or progression-free survival between the experimental and control groups. QoL=quality of life, NA=not available. Reference categories: QoL: reduction or NA; ESMO: low benefit; country: China.
